# Light sheet theta microscopy for rapid high-resolution imaging of large biological samples

**DOI:** 10.1186/s12915-018-0521-8

**Published:** 2018-05-29

**Authors:** Bianca Migliori, Malika S. Datta, Christophe Dupre, Mehmet C. Apak, Shoh Asano, Ruixuan Gao, Edward S. Boyden, Ola Hermanson, Rafael Yuste, Raju Tomer

**Affiliations:** 1 Department of Biological Sciences, Columbia University, New York, NY USA; 2 NeuroTechnology Center, Columbia University, New York, NY USA; 3 Data Science Institute, Columbia University, New York, NY USA; 4 Department of Neuroscience, Karolinska Institutet, Stockholm,, Sweden; 5 MIT Media Lab and McGovern Institute, Departments of Biological Engineering and Brain and Cognitive Sciences, MIT, Cambridge, MA USA; 6 Pfizer Internal Medicine Research Unit, Cambridge, MA 02139 USA

**Keywords:** Light sheet microscopy, Whole brain imaging, Quantitative imaging, *Hydra*, Calcium imaging, Tissue clearing, Expansion microscopy

## Abstract

**Background:**

Advances in tissue clearing and molecular labeling methods are enabling unprecedented optical access to large intact biological systems. These developments fuel the need for high-speed microscopy approaches to image large samples quantitatively and at high resolution. While light sheet microscopy (LSM), with its high planar imaging speed and low photo-bleaching, can be effective, scaling up to larger imaging volumes has been hindered by the use of orthogonal light sheet illumination.

**Results:**

To address this fundamental limitation, we have developed light sheet theta microscopy (LSTM), which uniformly illuminates samples from the same side as the detection objective, thereby eliminating limits on lateral dimensions without sacrificing the imaging resolution, depth, and speed. We present a detailed characterization of LSTM, and demonstrate its complementary advantages over LSM for rapid high-resolution quantitative imaging of large intact samples with high uniform quality.

**Conclusions:**

The reported LSTM approach is a significant step for the rapid high-resolution quantitative mapping of the structure and function of very large biological systems, such as a clarified thick coronal slab of human brain and uniformly expanded tissues, and also for rapid volumetric calcium imaging of highly motile animals, such as *Hydra*, undergoing non-isomorphic body shape changes.

**Electronic supplementary material:**

The online version of this article (10.1186/s12915-018-0521-8) contains supplementary material, which is available to authorized users.

## Background

Advances in tissue clearing methods [1] are enabling unhindered optical access to the structure and function of large intact biological systems such as mouse brain [2–5] and tumor biopsies [6]. Most of these approaches employ a cocktail of chemicals for cellular membrane lipid dissolution and/or refractive index smoothening to render the tissue transparent [1]. Furthermore, the development of physical tissue expansion approaches (expansion microscopy, ExM [7]) is enabling higher (super-resolution) effective imaging resolutions, although at the cost of ever-increasing sample sizes (up to 20-fold expansion demonstrated [8]). These approaches have the potential to accelerate discoveries across multiple domains of life sciences, including an understanding of the mammalian brain architecture, reconstructing tumor microenvironments, and in situ transcriptomics. However, taking full advantage of these techniques requires rapid high-resolution three-dimensional (3D) imaging of very large volumes.

**Fig. 1:**
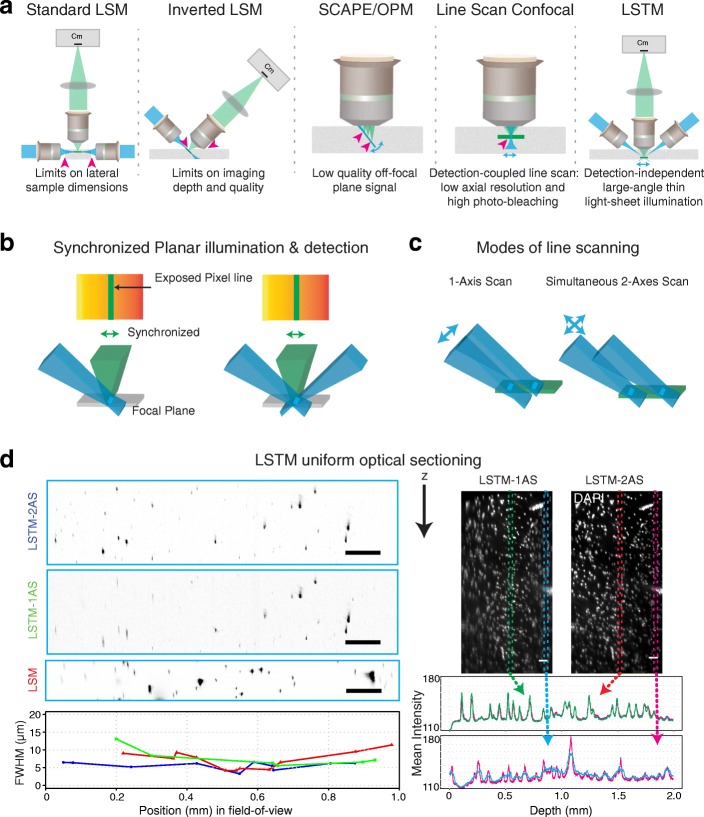
Light sheet theta microscopy (*LSTM*) for high-resolution quantitative imaging of large intact samples. **a** Light sheet microscopy (*LSM*) employs orthogonally illumination-detection optics, which limits the lateral dimensions of imaging volume. iSPIM, SCAPE/OPM and line scan confocal microscopy are partially effective in alleviating this limitation, however at the cost of reduction in usable working distance (*magenta arrowheads*) and image quality (e.g., SCAPE collects low-quality signal from non-native focal planes, and line scan confocal results in lower axial resolution and high photo-bleaching.). The proposed LSTM uses non-orthogonal (< 90°) illumination light sheets to effectively image very large samples, while maintaining high imaging speed and depth and uniform high resolution. **b** One or two light sheets intersect with the detection plane in a line illumination profile, which is synchronously scanned with the rolling shutter detection of an sCMOS camera to achieve optical sectioning. **c** Two scanning approaches: 1-axis scanning (*1-AS*) by perpendicular translation and simultaneous 2-axis scanning (*2-AS*, default LSTM) by translation along and perpendicular to the illumination axis such that the thinnest part is utilized for uniform planar illumination. **d** Comparison of point spread function (PSF) in 1-AS, default LSTM, and LSM configurations. *Left*: *x*-*z* maximum intensity projections of ~ 1 μm fluorescent microbeads imaged using the same detection (10×/0.6NA/8mmWD) and illumination (4×/0.28NA/28.5 mmWD) objectives. Axial full width at half maximum values (*FWHM*) across the field of view (*blue* LSTM in default 2-AS mode, *green* LSTM in 1-AS mode, *red* LSM). LSTM achieves uniform axial resolution (~ 4–6 μm FWHM) over the entire field of view, whereas both the 1-AS and LSM provide lower peripheral resolution (1-AS ~ 5–13 μm; LSM ~ 4–11 μm). *Right*: *x*-*z* projections (20 μm) of an image volume from a DAPI-stained human brain tissue. Additional file 5: Video 2 [29] provides 3D reconstructions. The graph compares the signal for a central and a peripheral region of interest. Scale bars: 100 μm

Conventional point-scanning approaches, such as confocal and two-photon microscopy, provide high imaging quality, but their slow imaging speeds and high photo-bleaching rates render them less effective for imaging of large volumes. Variants of confocal microscopy, including line scanning confocal microscopy (LSCM) [9, 10], can provide much higher imaging speeds due to parallel imaging of multiple points. However, these approaches still entail highly redundant illumination of out-of-focus parts of the samples and also reduced axial resolution and imaging depth, thus limiting their utility for high-resolution imaging of large cleared samples such as whole mouse brains (Fig. 1a). On the other hand, light sheet microscopy (LSM)-based approaches, with their orthogonal single-plane illumination and simultaneous whole-plane detection, are proving to be highly effective due to minimal photo-bleaching and high imaging speeds (2–3 orders of magnitude more than confocal) [11–13]. However, these advantages of orthogonal illumination-detection geometry also require unhindered optical access from the sides of the samples, thus limiting the lateral dimensions (along the illumination light sheet) of the imaging volumes (Fig. 1a). For example, we previously reported an optimized implementation of LSM, called CLARITY optimized light sheet microscopy (COLM) [5, 14], which allowed high-resolution imaging of entire intact mouse brains in a few hours imaging time, although with progressively reduced image quality towards the middle of the samples [5] due to the scattering of illumination light sheets. Similar attempts of LSM imaging of clarified rat brains resulted in much poorer image quality in large parts of the brain [15].

Alternative optical configurations of LSM have been explored to address these limitations, including the rotation of the illumination and the detection axes by 45° relative to the sample surface normal in objective-coupled planar illumination (OCPI) microscopy, inverted selective plane illumination microscopy (iSPIM), dual view iSPIM (diSPIM), and triple-view implementations [16–21], and the generation of illumination light sheets through the detection objectives themselves in swept confocally aligned planar excitation (SCAPE)/oblique plane microscopy (OPM) [22, 23]. The iSPIM/diSPIM approach does alleviate the limits on the lateral dimensions of imaging volumes, although at the cost of significant reduction in the usable working distance of the detection objective (Fig. 1a); therefore, it remains restricted to relatively low-numerical aperture (NA)/long-working distance (WD) detection objectives for imaging of large samples. The triple-view approach [21] incorporated an additional objective in the diSPIM implementation for simultaneously detecting the obliquely illuminated plane from the opposite side by rapid scanning with the piezo motors, resulting in enhanced spatial resolution for small samples (such as single cells). The SCAPE/OPM implementations use rotation optics to image an oblique plane illuminated using an oblique light sheet generated through the detection objective itself (Fig. 1a). The use of a single objective for detection as well as illumination is effective for fast volumetric imaging of small samples such as developing embryos. However, the imaging of an oblique (relative to the native detection focal plane) plane provides less-than-optimal image uniformity (across the imaged plane) and resolution. In summary, the scaling up of LSM imaging volumes, while maintaining uniform high imaging quality and speed, faces steep challenges. Here we address some of these challenges by developing a conceptually distinct microscopy framework, termed light sheet theta microscopy (LSTM), which builds upon the principles of LSM to allow high-speed quantitative imaging of large intact tissues with uniform high resolution. The LSTM uses two symmetrically arranged oblique light sheets, generated using independent illumination objectives, for rapid high-resolution imaging of large samples (Fig. 1). The oblique optical arrangement eliminates the restrictions on the sample lateral dimensions while ensuring high imaging speed and resolution and utilization of the entire available WD of high-NA detection objectives (Fig. 1a). We present a detailed characterization of the LSTM approach and demonstrate several real-world high-resolution imaging examples of very large samples including mouse and rat brain tissues, a large section of human brain, and a highly expanded ExM sample. The new capabilities of LSTM for high-speed quantitative imaging of larger samples at high resolution with low photo-bleaching may facilitate mapping of an entire post-mortem human brain (thick slab-by-slab) in a practical time-frame.

## Results

### LSTM implementation

LSTM includes a standard wide-field detection arm and two symmetrically arranged non-orthogonal (θ < 90°, relative to the detection axis) illumination arms for the generation of thin static light sheets that intersect at the detection focal plane (Fig. 1). The resulting thin line illumination profile is scanned along the detection focal plane in synchrony with the row-by-row rolling shutter imaging with a scientific complementary metal-oxide-semiconductor (sCMOS) camera (virtual slit effect [5]) to achieve thin optical sectioning (Fig. 1b). This is realized by simultaneously translating the light sheet along (using an electrically tunable lens (ETL)) and perpendicular (using a galvanometer (galvo) scanner) to its propagation direction such that only the thinnest part intersects the detection plane (Fig. 1c). Note that the translation of light sheets along their propagation direction may also be achieved using alternative implementations, including fast piezo motors for translating illumination objectives, or using an acoustic tunable lens such as the tunable acoustic gradient index of refraction (TAG) lens (TAG optics) [14, 24–28].

**Fig. 2:**
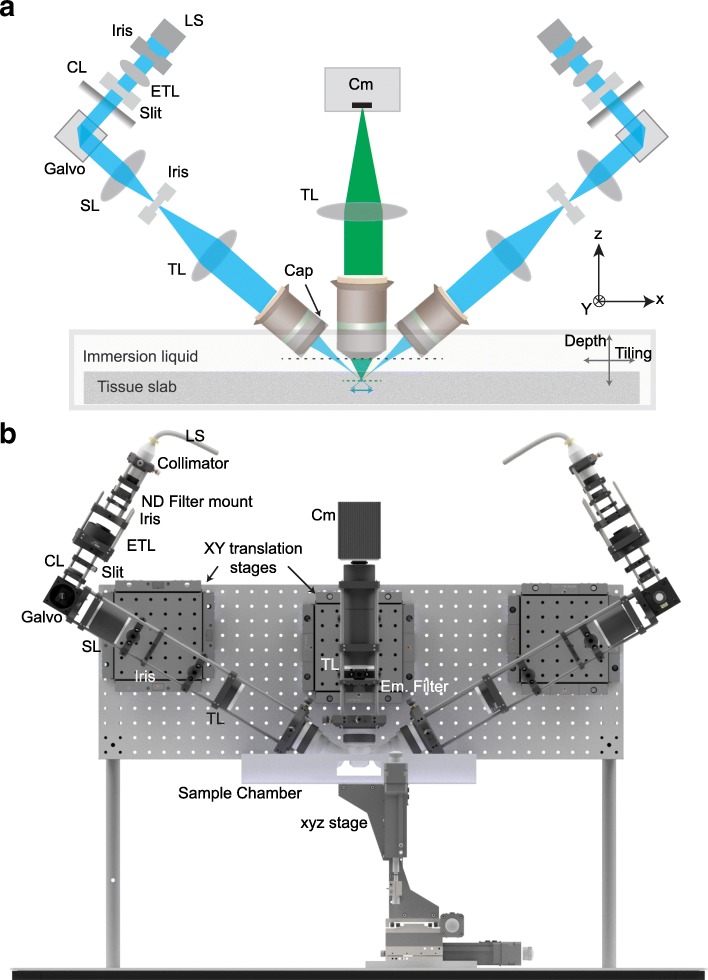
LSTM microscopy implementation. **a** LSTM optical path. Two symmetric light sheets are generated by using a cylindrical lens (*CL*), scan lens (*SL*), tube lens (*TL*), and illumination objectives. The galvo scanners are used to translate the light sheets perpendicular to their propagation direction, and the electrically tunable lens (*ETL*) for translating the thinnest part of the light sheets along the propagation direction. An input beam of ~ 10 mm diameter is then trimmed through an iris. A slit is placed after the ETL to control the effective numerical aperture of the illumination. An additional iris is placed between the SL and TL to control the light sheet width. The illumination axes are arranged at ~ 60° to the detection axis. A custom 3D-printed cap with a quartz coverslip is attached to the illumination objective to allow dipping in the immersion oil to ensure that the low NA illumination rays from an air illumination objective enter perpendicularly to the oil. The detection arm consists of a detection objective (Olympus 10×/0.6NA/8mmWD or 25×/1.0NA/8mmWD, both with correction collars), a tube lens, and an sCMOS camera. **b** 3D model of the LSTM microscope. A vertical breadboard was used to mount the caged optical assemblies via *x*-*y* manual translation stages to allow fine adjustments. A sample chamber was attached to a 3-axis (*x*, *y*, *z*) motorized stage assembly. See also Additional files 1, 2, 3, and Additional file 4: Video 1 for further details and Table 1 for complete parts list

The LSTM illumination and detection arms were implemented as rigid assemblies (using a caging system from Thorlabs) which were connected to a vertically mounted breadboard via *x*-*y* manual translation stages for finer adjustments (Fig. 2, Additional file 1: Figure S1, Additional file 2: Figure S2, Additional file 3: Figure S3, Additional file 4: Video 1). An open-top sample mounting strategy was implemented by using a custom 3D-printed sample chamber (Fig. 2, Additional file 1: Figure S1) attached to a high-accuracy *x*-*y*-*z* motorized stage assembly (LNR50S, Thorlabs). The imaging samples can be mounted in a quartz glass cuvette of appropriate size. The illumination arm consists of a laser source (SOLE-6, 405, 488, 561, and 647 nm, Omicron-Laserage), a collimator (~ 10-mm beam diameter, Omicron-Laserage), an ETL (Optotune), a cylindrical lens (LJ1695RM-A, *f* = 50 mm, Thorlabs), a galvo scanner (GVS001, Thorlabs), a scan lens (CLS-SL, Thorlabs), a tube lens (*f* = 200 mm, ITL200, Thorlabs), and an illumination objective (Macro 4×/0.28NA/28.5mmWD, Olympus; the arrangement of different components is summarized in Additional file 2: Figure S2, and the parts list is provided in Table 1). Note that even though the ETLs are mounted at an oblique angle (as opposed to vertical), this does not result in any significant observable aberrations because of the low NA of the illumination objectives. In addition, an iris is placed after the collimator to remove peripheral spreads of Gaussian beams, a one-dimensional (1D) slit is positioned before the cylindrical lens to control the effective NA (hence the light sheet thickness), and a second iris is placed at the conjugate plane between the scan lens and tube lens to control the light sheet width (i.e., the dimension of light sheet perpendicular to its propagation direction). The detection arm is composed of a detection objective (Olympus 10×/0.6NA/8mmWD or 25×/1.0NA/8mmWD, both with refractive index correction collars, from water to oil), a multi-band emission filter (FF01-432/515/595/730-50-D, Semrock), a tube lens (*f* = 200 mm, ITL200, Thorlabs), and an sCMOS camera (Orca Flash 4.0, Hamamatsu; 2048 × 2048 pixels, 6.5 μm × 6.5 μm pixel size). Note that since we used a 200-mm tube lens with Olympus objectives, the effective magnifications are 11.11 and 27.78 for the 10× and 25× objectives respectively. The LSTM assembly was optically aligned by placing a prism mirror (with scratches in the center, see Additional file 1: Figure S1 for mounting arrangements) in the focal plane of the detection optics, to visualize the location and cross section of the light sheet relative to the detection focal plane. The light sheet positioning parameters (i.e., galvo and ETL) were optimized such that the thinnest part was in alignment with the center of the field of view of the detection plane. Next, fluorescent beads, embedded in a high concentration (> 2%, to restrict the signal source to the gel surface) agarose gel, were used to find the optimal galvo and ETL parameters by examining the extent and focus quality. All imaging experiments were performed using 405 (for 4’,6-diamidino-2-phenylindole (DAPI)) or 488 nm (for *eYFP* detection) laser lines. LSTM parameters were adjusted to use a 2 to 5 μm effective light sheet thickness.

**Table 1:** Parts list of LSTM

Vendor	Number	Qty.	Description
Detection unit			
Thorlabs	CXY2	1	60-mm Cage System Translating Lens Mount for Ø2” Optics
Thorlabs	LCP90F	1	60-mm Removable Cage Plate
Thorlabs	SM2A20	1	SM2-M38 Adapter for Nikon Tube Lens
Thorlabs	SM2L30	2	SM2 Lens Tube, 3” Thread Depth, One Retaining Ring Included
Thorlabs	LCP09	2	60-mm Cage Plate with Ø2.2″ Double Bore for SM2 Lens Tube Mounting
Thorlabs	ER10	4	Cage Assembly Rod, 10″ Long, Ø6 mm
Thorlabs	SM1A1	1	Adapter with External SM05 Threads and Internal SM1 Threads
Thorlabs	SM2A31	1	Adapter with External C-Mount Threads and Internal SM2 Threads
Hamamatsu	C13440	1	sCMOS Orca Flash 4.0 V3.0 camera
/	Objective Adapter	1	Custom-made Adapter from SM2 to M34 threading
Thorlabs	SM2V10	1	Ø2” Adjustable Lens Tube, 0.81″ Travel
Olympus	XLPLN10XSVMP	1	10× Long Working Distance Detection Objective
Olympus	XLSLPLN25XGMP	1	25× Long Working Distance Detection Objective
Thorlabs	LCP01B	2	60-mm Cage Mounting Bracket
Thorlabs	RSH2	2	Ø1” Post Holder with Flexure Lock, Pedestal Base, L = 2”
Thorlabs	RS2	2	Ø1” Pillar Post, 1/4″-20 Taps, L = 2″, 8–32 Adapter Included
Thorlabs	TBB0606	2	Large-Area Translation Stage, 6″ × 7.66”
Thorlabs	TTL200	1	*f* = 200 mm Tube Lenses for Wide Field Imaging
Semrock	FF01-432/515/595/730-50-D	1	Multi-Band Emission Filter
Illumination unit			
Thorlabs	SM2V10	2	Ø2” Adjustable Lens Tube, 0.81″ Travel
/	Objective Adapter	2	Custom-made Adapter from SM2 to M34 Threading
Olympus	XLFLUOR4X/340	2	4× Air Objective
Thorlabs	SM2A20	2	SM2-M38 Adapter for Nikon Tube Lens
Thorlabs	CXY2	2	60-mm Cage System Translating Lens Mount for Ø2” Optics
Thorlabs	SM2A31	2	Adapter with External C-Mount Threads and Internal SM2 Threads
Thorlabs	SM2V10	2	Ø2" Adjustable Lens Tube, 0.81" Travel
Thorlabs	LCP09	2	60-mm Cage Plate with Ø2.2” Double Bore for SM2 Lens Tube Mounting
Thorlabs	LCP01B	4	60-mm Cage Mounting Bracket
Thorlabs	RS2	4	Ø1” Pillar Post, 1/4″-20 Taps, L = 2″, 8–32 Adapter Included
Thorlabs	RSH1.5	4	Ø1” Post Holder with Flexure Lock, Pedestal Base, L = 1.5”
Thorlabs	TBB0606	4	Large-Area Translation Stage, 6″ × 7.66”
Thorlabs	ER05	8	Cage Assembly Rod, 1/2″ Long, Ø6 mm
Thorlabs	LCP02	6	30-mm to 60-mm Cage Plate Adapter, 8–32 Tap
Thorlabs	LJ1695RM-A	2	Ø1”, N-BK7 Mounted Plano-Convex Round Cylindrical Lens
Thorlabs	CRM1L	2	Cage Rotation Mount for Ø1” Optics, Double Bored with Setscrew, 8–32 Tap
Thorlabs	CP20S	2	30-mm Cage System Iris, Ø20.0-mm Maximum Aperture
Thorlabs	CP90F	2	30-mm Removable Cage Plate, Front and Back Plate, Internal SM1 Threading
Thorlabs	CXY1	2	30-mm Cage System, XY Translating Lens Mount for Ø1” Optics
Thorlabs	CP12	2	30-mm Cage Plate, Ø1.2″ Double Bore for SM1 Lens Tube Mounting
Thorlabs	LCP01	4	60-mm Cage Plate, SM2 Threads, 0.5” Thick, 8–32 Tap (Two SM2RR Retaining Rings Included)
Thorlabs	CLS-SL	2	Scan Lens with Large Field of View, 400 to 750 nm, EFL = 70 mm
Thorlabs	ER18	6	Cage Assembly Rod, 18″ Long, Ø6 mm
Thorlabs	LCP50S	2	60-mm Cage System Iris, Ø50.0 mm Maximum Aperture
Optotune	EL-16-40-TC	2	Electrically Tunable Lens
Thorlabs	ER4	18	Cage Assembly Rod, 4″ Long, Ø6 mm
Thorlabs	VA100C	2	30-mm Cage System Adjustable Slit, 8–32 Tap, Imperial Micrometer
Thorlabs	GVS001	2	1D Galvo System, Silver-Coated Mirror, PSU Not Included
Thorlabs	GCM001	2	1D Galvo 30-mm Cage System Mount
Omicron	/	2	Collimator with ~ 10-mm Bead Diameter Output (Custom-made)
Omicron	SOLE-6	1	SOLE-6 Engine Containing Four Laser Lines: 405, 488, 561, 647 nm
Thorlabs	TTL200	2	*f* = 200 mm Tube Lenses for Wide Field Imaging
Base support			
Thorlabs	MB1236	1	Aluminum Breadboard 12″ × 36″ × 1/2″, 1/4″-20 Taps
Thorlabs	RS12	4	Ø1” Pillar Post, 1/4″-20 Taps, L = 12″, 8–32 Adapter Included
Thorlabs	C1001	4	Post Mounting Clamp for Ø1” Post
Stage and sample mounting			
Thorlabs	LNR50S	3	50-mm (1.97″) TravelMax Translation Stage, 1/4″-20 Taps
Thorlabs	LNR50P3	1	XY Adapter Plate for LNR50 TravelMax Stages, Imperial Hole Spacings
Thorlabs	LNR50P2	2	Right-Angle Bracket for LNR50 TravelMax Stages, Imperial Threads
/	Theta chamber	1	Custom-made 3D Printed Sample Chamber
Controls and electronics			
National Instruments	CA1000	4	Configurable Connector Accessory Enclosure
National Instruments	NI PXIe-1082	1	Modular Electronic Instrumentation Platform
Thorlabs	GPS011	1	Galvo System Linear Power Supply
Thorlabs	LEDD1B	2	T-Cube LED Driver with Trigger Mode, 1200 mA
Thorlabs	BSC203	1	BSC203 - Three-Channel APT™ Benchtop Stepper Motor Controller
Dual Xeon Workstation	/	1	Custom Workstation with Supermicro X10DRHCT Motherboard

**Fig. 3:**
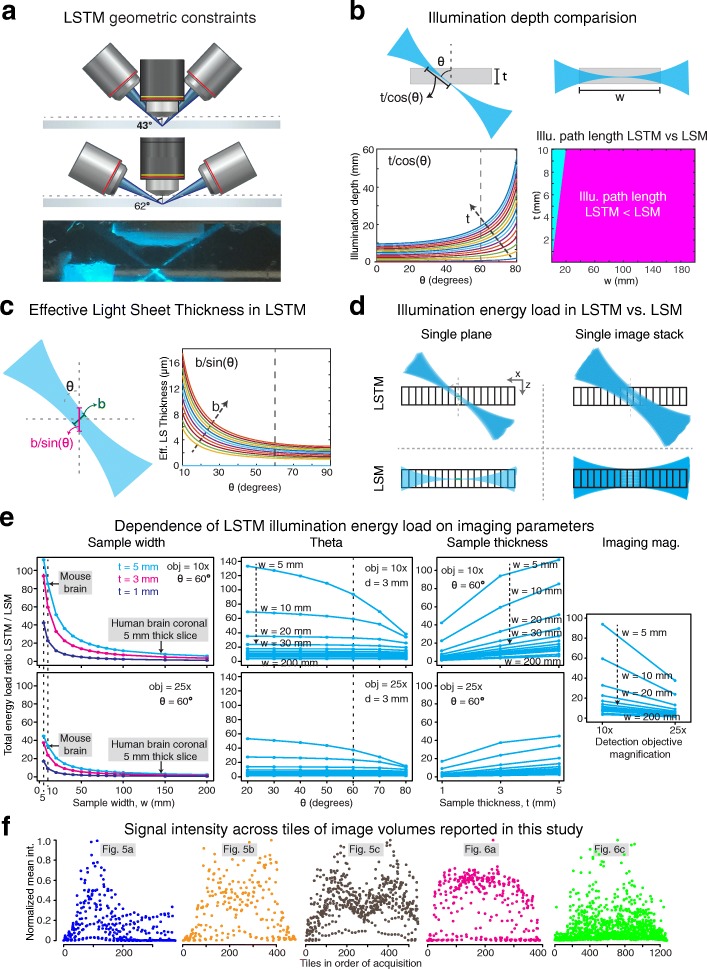
LSTM characterization. **a** Geometric constraints in LSTM. Specific example of using Olympus 4×/0.28NA/29.5WD and 10×/0.6NA/8mmWD for illumination-detection. Note that the working distance of the air illumination objective is elongated in high refractive index immersion media (Additional file 3: Figure S3). Angular separation of ~ 60° was used for all experiments. **b** Comparison of maximum illumination path length (MIPL) required for full sample coverage in LSTM and LSM. The illumination light sheets need to penetrate the entire width (*w*) of the sample (or half width for two-sided illumination) in LSM, whereas MIPL depends on the angular arrangement and the tissue thickness (*t*) in LSTM. *Bottom left*: dependence of LSTM MIPL on θ and sample thickness (*t*, *arrow* indicates increasing *t*). *Bottom right*: MIPL dependence on the sample width and thickness: *magenta* and *cyan* highlight LSTM < LSM and LSTM > LSM respectively, assuming θ = 60° (see Additional file 6: Figure S4 for full θ range). **c** Effective planar illumination thickness can be approximated as *b*/sin(θ), where *b* is the actual light sheet thickness. The *right graph* plots the effective light sheet thickness as a function of θ and *b* (*arrow* points to increasing *b*). **d** Comparison of redundant illumination in LSTM and LSM for imaging of a single plane (*top row*) and an image stack (*bottom row*). **e** Ratios of total illumination energy loads (LSTM/LSM) as a function of sample width (*w*), angular configuration (θ), sample thickness (*t*), and objective magnification (10× and 25×). Illumination energy load is higher in LSTM for smaller samples and similar to LSM for larger samples. The energy load ratio also decreases with increased angular separation (60° is marked) and the magnification of detection objective. Additional file 7: Figure S5 provides details. **f** The average signal of tiles in the order of acquisitions. Note that no significant photo-bleaching trend is observed

The angular separation of the illumination and detection arms is constrained by the physical dimensions and optical properties of the detection and illumination objectives. For instance, only an angular separation range of 43–62^°^ is feasible for the specific combination of illumination (Macro 4×/0.28NA/29.5mmWD, Olympus) and detection objectives (10×/0.6NA/8mmWD or 25×/1.0NA/8mmWD, Olympus) used in this study (see Fig. 3a, Additional file 3: Figure S3). Note that the WD of the illumination objective is specified for use in air; therefore, an approximate effective WD in immersion oil was calculated as shown in Additional file 3: Figure S3.

### LSTM characterization

**Fig. 4:**
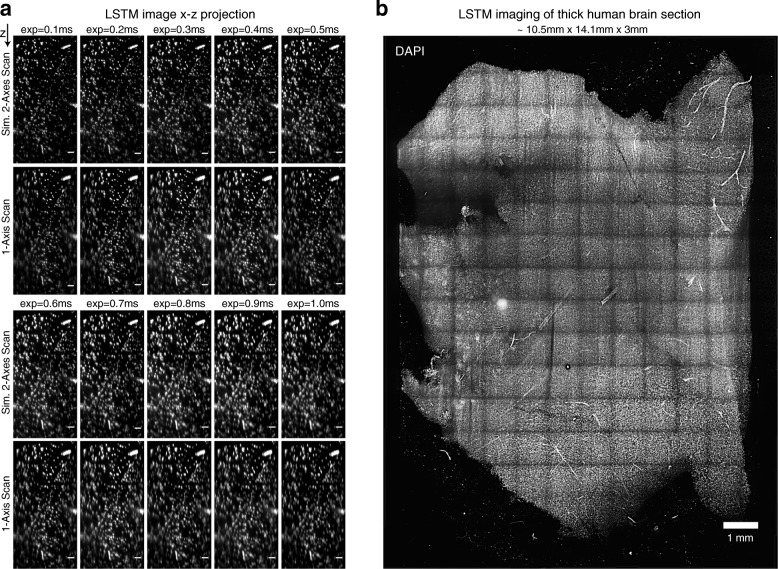
LSTM optical sectioning. **a** *x*-*z* maximum intensity projections of an image stack acquired from human brain tissue, shown in (**b**), stained with DAPI. The camera rolling shutter exposure time determines the effective slit (rolling shutter) width (0.1–1 ms, i.e., 66–665 μm on the sCMOS sensor and 6–60 μm on the sample. The images were acquired using two different scanning modes: LSTM 1-axis scan (1-AS) and LSTM 2-axis scan (2-AS, default). Total frame exposure was 20 ms for all the images. As evident, the 2-AS mode allows for uniform planar illumination for achieving quantitative imaging, and the axial resolution decreases with increased rolling shutter exposure. All scale bars are 100 μm. **b** LSTM imaging of a large thick section of cleared human brain tissue (~ 10.5 mm × 14.1 mm × 3 mm) stained with DAPI. We used 0.5-ms rolling shutter exposure settings and 20 ms for entire frame exposure to acquire this dataset. Scale bar is 1 mm

We first characterized the LSTM point spread function (PSF) by imaging micrometer-size fluorescent microbeads. The same microbeads were also imaged with LSM as well as a non-optimal 1-axis LSTM scanning procedure (i.e., the light sheet is only translated perpendicular to its propagation; marked as 1-AS mode in Fig. 1c), using the exact same detection (10×/0.6NA) and illumination objectives (4×/0.28NA). The quantification and comparison of full width at half maximum (FWHM, Fig. 1d) revealed that LSTM indeed allows for uniform high axial resolution (~ 4 to 6 μm for the combination of these objectives) across the entire field of view, whereas as expected, both the LSM and the non-optimal 1-AS LSTM scan procedure resulted in lower axial resolution on the peripheries of the field of view (> 11 μm) (Fig. 1d, Fig. 4 and Additional file 5: Video 2 [29]). Next, we compared the maximum illumination path length (MIPL), i.e., the maximum distance the illumination light sheets need to penetrate inside the tissue to achieve complete sample coverage. The shorter the illumination path length, the lesser the scattering, hence potentially better the imaging performance. In LSM, the MIPL depends on the sample width as the light sheet needs to penetrate the entire width (half width for two-sided illumination) of the sample to achieve complete coverage, whereas in LSTM, the MIPL depends on the angular separation of the illumination-detection arms and the tissue thickness (*t*): *t*/cos(θ) (Fig. 3b). The ratio of LSTM and LSM illumination path lengths was calculated for varying sample dimensions and the θ values (Fig. 3b shows θ = 60°, and Additional file 6: Figure S4 shows θ = 0°, 10°, 20°, 30°, 40°, 50°, 60°, 70°, and 80°). This revealed that the LSTM illumination path length was smaller than that for LSM for wider samples, and larger for smaller samples, suggesting the complementary advantages of LSTM and LSM for high-resolution imaging of large and small samples respectively. Since the LSTM illumination path length decreases with decreasing angular separation (*t*/cos(θ)), minimizing the angular separation (θ) will result in potentially higher imaging quality; however, the angular separation also affects the effective light sheet thickness (approximated as *b*/sin(θ), Fig. 3c) in an inverse relationship. Therefore, we used the maximum allowed angular separation (~ 60°) to achieve better axial resolution.

Further, we sought to assess and compare the total energy loads in LSTM vs. LSM imaging (Fig. 3d–f, Additional file 7: Figure S5). Since LSTM employs only the thinnest part of the illumination light sheet for imaging, this results in significant redundancy in illumination. Similarly, in LSM, the imaging of large samples (i.e., larger than a single field of view of detection) entails redundant illumination of the parts of samples (along the illumination) not being imaged. As summarized in the Fig. 3d schematics, the total redundant energy load depends on the sample thickness in LSTM (Fig. 3d, top row), and on the sample width in LSM (Fig. 3d, bottom row). Therefore, for a quantitative comparison of LSTM vs. LSM energy loads, we calculated the ratio of the total energy loads (the procedure is summarized in Additional file 7: Figure S5) as a function of the sample width, thickness, angular separation (θ) of the LSTM, and the detection objective magnification. Note that we are comparing the LSTM energy load with the scanned light sheet microscopy, which is the commonly used implementation for imaging of large samples (e.g., COLM [5]) due to the reduced coherent illumination scattering and the synchronization possibilities with the rolling shutter detection of sCMOS cameras. Therefore, the dwell time of the illumination line profile is the same for both. As summarized in Fig. 3e, the LSTM indeed imparts a much higher energy load for imaging of smaller samples, but the ratio approaches 1 with increasing sample size and higher detection magnification. The LSTM energy load also depends on the angular separation (lower for larger θ); θ = 60° was used for these calculations. To complement these calculations, we also performed empirical assessment of the signal photo-bleaching for the LSTM imaging datasets reported in this study. As shown in Fig. 3f, no significant trend is observed, suggesting minimal photo-bleaching consequences. In summary, the LSTM total energy load is much higher for smaller samples, but is comparable to LSM for high-resolution imaging of larger samples, further supporting the overall complementary advantages of LSTM and LSM for imaging of larger and smaller samples. Next, we characterized the effect of the width of the rolling shutter (i.e., the “virtual” slit, controlled by the rolling shutter exposure parameter). As expected, the axial resolution is better for smaller rolling shutter widths (Fig. 4a), analogous to the effect of the pinhole diameter in confocal microscopy.

Finally, a major advantage of LSM is the high imaging speed. Similar to standard LSM implementations (e.g., COLM [5, 14]), the LSTM imaging procedure involves synchronization of the illumination line with the rolling shutter detection of the sCMOS camera. Therefore, a single image acquisition takes 20 ms (which is a property of the camera imaging speed in rolling shutter mode), yielding 50 full frames (2048 × 2048) per second. The LSTM also allows the synchronization of the two illumination line profiles independently with the bi-directional readout mode of the sCMOS sensors (10 ms for full frame). However, the imaging of large volumes requires the use of long travel-range motorized stages, which are typically the rate-limiting step in the entire imaging procedure. For example, as reported previously [5], a typical sample stage takes more than 50 ms for a 5-μm *z*-step sample motion, resulting in ~ 60–70 ms acquisition time per *z*-plane, i.e., 10–15 Hz imaging speed for LSTM as well as state-of-the-art LSM implementations for large sample imaging. Alternatively, instead of using a step-wise motion, the sample can also be continuously scanned at a small uniform speed to allow overall higher imaging speeds, although at the cost of significant shearing artifacts. We would also like to highlight that, unlike LSM (e.g., the COLM system [5]), LSTM imaging did not require any pre-calibration/adaptive parameter correction steps because of the overall smaller illumination path lengths, resulting in a higher effective (about twofold) imaging speed.

### LSTM enables rapid quantitative imaging of large samples with uniform high resolution

**Fig. 5:**
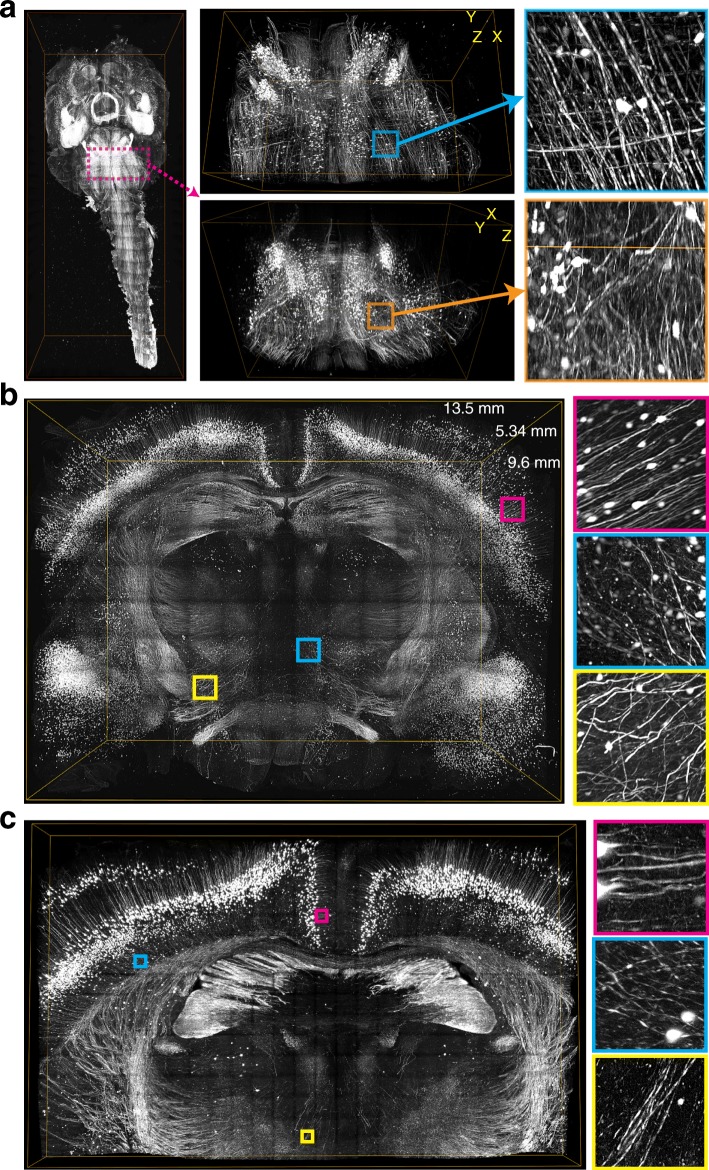
Rapid uniform high-resolution imaging of mouse central nervous system. **a** A CLARITY-cleared *Thy1-eYFP* transgenic mouse brain with attached spinal cord was imaged with LSTM microscopy using 10×/0.6NA/8mmWD detection objective (correction collar adjusted to 1.45 refractive index). A rolling shutter exposure of 0.5 ms and a full frame exposure of 20 ms were used. High-resolution 3D rendering was generated after 2 × 2-fold down-sampling. The bounding boxes are 11.8 mm × 27.6 mm × 5.2 mm for the whole sample and 5.1 mm × 3.1 mm × 3.5 mm for the subvolume (*magenta*). Images were acquired with 5-μm *z*-spacing using an effective light sheet thickness of ~ 5 μm. Lateral pixel sampling was 0.585 × 0.585 μm. A detailed volume rendering is shown in Additional file 8**:** Video 3 [29]. **b** A large thick coronal slice of a *Thy1-eYFP* transgenic mouse brain was imaged with LSTM using 488 nm excitation wavelength. A rolling shutter exposure window of 0.5 ms and a full frame exposure of 20 ms were used. The volume rendering was performed using 4 × 4 fold down-sampled data. The bounding box is 9.6 mm × 13.5 mm × 5.34 mm. Images were acquired with 5-μm *z*-spacing using an effective light sheet thickness of ~ 5 μm. Lateral pixel sampling was 0.585 × 0.585 μm. Additional file 9: Video 4 [29] shows volumetric rendering. **c** The same sample as shown in **b** was imaged with a high-NA 25×/1.0NA/8mmWD objective. A rolling shutter exposure window of 0.4 ms and a full frame exposure of 20 ms were used. The volume rendering was performed after 2 × 2-fold down-sampling. The bounding box is 6 mm × 9.6 mm × 0.5 mm. Images were acquired with 5-μm *z*-spacing using an effective light sheet thickness of ~ 3 μm. Lateral pixel sampling was 0.234 × 0.234 μm

**Fig. 6:**
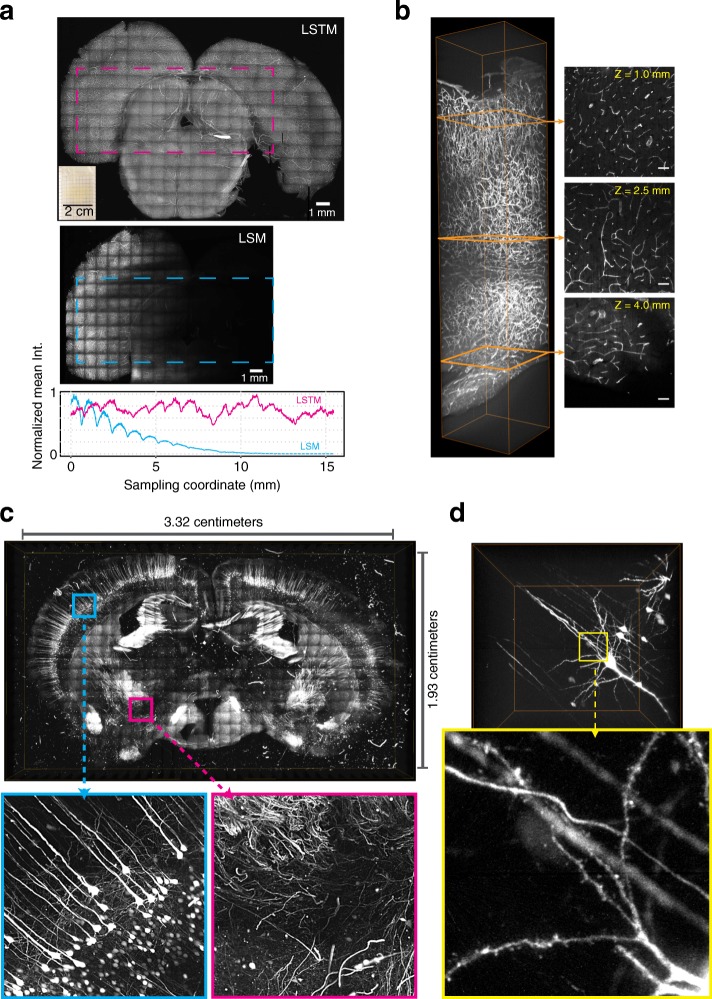
LSTM enables rapid uniform high-resolution imaging of very large samples. **a** For an unbiased comparison of the imaging performance of LSTM and LSM a highly cleared large rat brain tissue (~ 2 cm wide and ~ 5 mm deep; vasculature stained with tomato lectin) was imaged using the exact same detection (10×/0.6NA/8mmWD, correction collar adjusted to 1.45 refractive index) and illumination objectives (4×/0.28NA/28.5WD). Maximum intensity projections are shown. The *bottom graph* profiles the mean intensity across the length of the specified (*dashed rectangles*) regions of interest. In LSM (*cyan*), the intensity signal is progressively degraded towards the interior of the sample, whereas LSTM (*magenta*) allows uniform quality across the entire sample. The scale bars are 1 mm. **b** An image stack from the sample shown in **a**. Maximum intensity projections (50 μm) are shown at three different depths (*orange*). The bounding box is 1 mm × 1 mm × 5 mm. The scale bars are 100 μm. A detailed volume rendering is shown in Additional file 10: Video 5 [29]. **c** Uniformly expanded (~ 4-fold in all three dimensions) slice of *Thy1-eYFP* transgenic mouse was imaged using LSTM with 10×/0.6NA/8mmWD detection objective. A rolling shutter exposure window of 0.2 ms and a full frame exposure of 20 ms were used. The resulting dataset consists of 723,200 images (2048 × 2048 pixels) and required ~ 22 h of acquisition time. The volume rendering was performed with 8 × 8 fold down-sampled dataset. Zoomed-in images are marked. **d** An image stack from the dataset shown in **c**. The bounding box size is 1.2 mm × 1.2 mm × 1 mm. Note that the dendritic spines can be unambiguously identified. Detailed volume rendering in Additional file 11: Video 6 [29]

To assess the performance of LSTM in real-world experimental scenarios, we performed high-resolution imaging of cleared samples of various sizes and shapes. First, we imaged a large thick section of cleared DAPI-stained human brain (~ 10.5 mm × 14.1 mm × 3 mm) and a cleared intact central nervous system (11.8 mm × 27.6 mm × 5.2 mm) of a *Thy1-eYFP* transgenic mouse using 405 nm and 488 nm illumination wavelengths respectively. As shown in Figs. 4b and 5a and Additional file 8: Video 3 [29], LSTM enabled rapid high-resolution imaging of these large samples with uniform imaging quality throughout, even for a sample with highly curved surfaces. We further imaged a thick (~ 9.6 mm × 13.5 mm × 5.34 mm; the sample expanded ~ 1.5- to 2-fold due to the immersion in glycerol solution [14]) coronal slice of a CLARITY-cleared *Thy1-eYFP* transgenic mouse brain, with 10×/0.6NA/8mmWD (Fig. 5b, Additional file 9: Video 4 [29]) as well as 25×/1.0NA/8mmWD (Fig. 5c) objectives. As demonstrated by zoom-in views at various locations in the samples, LSTM provides high uniform quality throughout the sample. To directly compare the performance of LSTM with LSM, we also imaged a very wide (~ 1.5 cm) and thick (> 5 mm, i.e., as thick as mouse brain) slice of highly cleared (see Fig. 6a) rat brain, which was stained for uniform distributed blood vessels (Fig. 6a; using tomato lectin, excitation wavelength 488 nm). Previous attempts of using LSM for the imaging of rat brain resulted in very poor image quality in most of the internal parts of the brain [15] because of the heavy scattering of illumination light sheets. As shown in Fig. 6 and Additional file 10: Video 5 [29], LSTM allowed uniform high-resolution imaging of the entire tissue, whereas LSM resulted in progressively poor image quality towards the center of the sample, similar to the previous report [15].

To further demonstrate the suitability of LSTM for imaging of very large samples, we performed rapid high-resolution imaging of a very large (3.32 cm × 1.93 cm × 1 mm) uniformly expanded (using the ExM approach [7]) brain slice of a transgenic *Thy1-eYFP* mouse using 488 nm excitation wavelength (Fig. 6c, d, Additional file 11: Video 6 [29]). The advent of tissue expansion approaches (ExM) is enabling higher effective imaging resolution, however at the cost of hugely increased imaging time and data sizes. For example, the imaging of this sample with state-of-the-art confocal or two-photon microscopy will likely take several weeks of continuous imaging, whereas LSTM took only ~ 22 h (using 10×/0.6NA), yielding 723,200 full frame (2048 × 2048 pixels) images. The resulting dataset reveals the finest details of brain neuronal architecture (e.g., dendritic spines, Fig. 6c, d and Additional file 11: Video 6 [29]). Taken together, these imaging examples clearly demonstrate the suitability of LSTM for rapid high-resolution imaging of very large samples of different shapes. Unlike LSM, LSTM provides high uniform imaging quality even for the interior parts of the samples. In summary, these examples clearly demonstrate the complementary advantages of LSTM over LSM for rapid high-resolution imaging of large samples.

### LSTM enables rapid imaging of nervous system-wide neuronal dynamics of freely motile animal

**Fig. 7:**
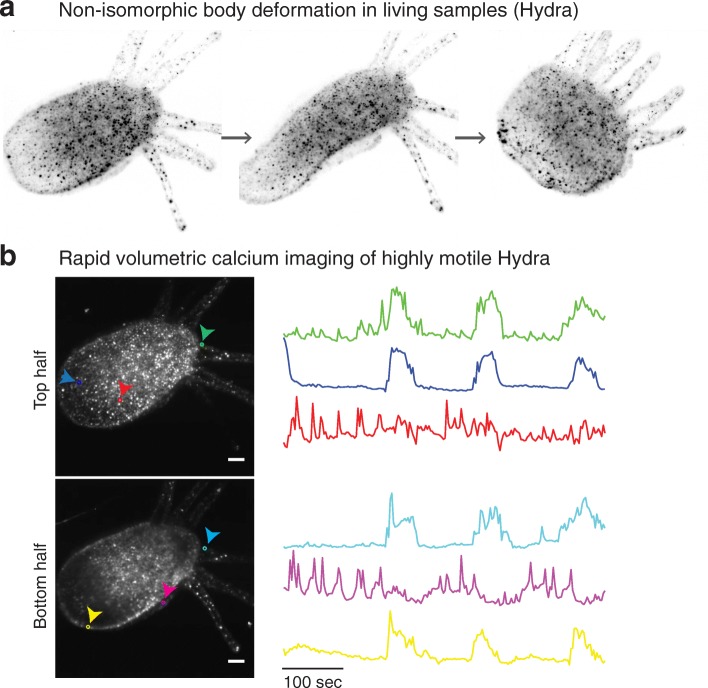
LSTM enables rapid volumetric imaging of highly motile animals. Live samples can undergo substantial non-isomorphic rearrangements in their body shape and cellular density, resulting in continuously changing local optical properties. LSM is particularly susceptible to misalignments and other aberrations because of the use of orthogonal light sheet illumination. LSTM is uniquely suitable for rapid volumetric live imaging of such difficult samples, as demonstrated by imaging of highly motile *Hydra*. **a** *Hydra* image is shown at different time points to highlight the non-isomorphic changes in freely moving animal. **b** LSTM was used to perform long-term (> 1 h demonstrated, Additional file 12: Video 7 [29]) high-resolution live imaging of an adult *Hydra* expressing GCaMP6s [31]. Each volume consists of 17 *z*-planes. Manual tracking and analyses of calcium signaling were performed for the first ~ 500 s of recording. Maximum intensity projections covering the two halves are shown. Representative neuronal traces are shown for cells marked in corresponding colors. As shown in Additional file 13: Video 8 [29], the neuronal traces correlate with the rapid longitudinal contraction behavior of *Hydra*, and the other two traces are part of rhythmic potential circuits, in excellent agreement with the observations reported recently [31]. Scale bars are 100 μm

Finally, we demonstrate the compatibility of LSTM in capturing the nervous system dynamics of a highly motile animal. Live samples often undergo substantial rearrangements in their body shape and cellular density which significantly alter their local optical properties. Although LSM-based imaging methods have been effective in capturing the cellular dynamics of developing embryos and the neuronal activity of immobilized zebrafish larvae, LSM remains susceptible to large changes in shape and density of motile samples, mainly because of the use of orthogonal illumination. This limitation has been partly addressed by utilizing a sophisticated array of hardware and software components that facilitate real-time adaptation of light sheet parameters [30]. LSTM, with its non-orthogonal illumination, provides a simpler and highly effective solution. We tested this hypothesis by performing rapid volumetric calcium imaging of a highly motile *Hydra*, which has been recently established as an effective model for exploring the role of neuronal circuit activity on behavior [31, 32]. We found that, indeed, LSTM enables aberration-free calcium imaging of freely behaving *Hydra* undergoing drastic changes in body shape and cellular density in the recordings (Fig. 7a and Additional file 12: Video 7, Additional file 13: Video 8 [29]). In a way, the large and non-isomorphic body deformation of *Hydra* represents the worst-case scenario for tracking the activity of neurons during behavior. We validated LSTM datasets by extracting and comparing neuronal traces with previous observations, finding excellent agreement [31]. Note that, for this demonstration, we used the relatively slow process of step-wise motion of the sample stage to acquire the image stacks. The LSTM mechanism can be straightforwardly combined with piezo motor-based synchronous rapid scanning of the detection objective and also with extended detection depth of field [14].

## Discussion

We reported the development of LSTM, which addresses the lateral size limitation of state-of-the-art LSM approaches. LSTM employs two symmetrically arranged oblique static light sheets generated using independent illumination objectives, and their scanning using simultaneous two-dimensional (2D) translation along (using an electrically tunable lens (ETL)) and perpendicular (using galvo scanners) to their propagation directions, resulting in uniform illumination and detection (using synchronized rolling shutter detection of sCMOS cameras) of thin optical sections. This optical configuration eliminates the fundamental restrictions of LSM on the lateral dimensions of the imaging volumes while ensuring high imaging speed and resolution and the utilization of the entire available WD of high-NA detection objectives. The use of two light sheets (as opposed to one) ensures better quality (e.g., if one of the sheets is obstructed by opaque objects) but is not necessary. To minimize optical aberrations, we used refractive index optimized detection objectives and used 3D-printed caps (with quartz coverslips) for illumination objectives (air, low NA, and long WD) to ensure a perpendicular incidence of light sheets to the mounting media.

The enhanced performance of LSTM entails an increased overall energy load for imaging of smaller samples, such as embryos, but remains comparably as low as LSM for high-resolution imaging of large samples, as supported by the simulations (Fig. 3d, e) and empirical calculations (Fig. 3f). Moreover, the use of only the thinnest part of the light sheets enables quantitatively uniform illumination of the entire detection plane, which is a foremost requirement for 3D quantitative imaging. Therefore, LSTM is most suitable for rapid high-resolution imaging of very large samples, whereas LSM provides better performance for smaller samples.

The use of ETLs in the illumination arm for translating light sheets along their propagation direction introduces an additional component which needs to match the camera acquisition speed. The high-resolution imaging of large samples essentially requires full camera frame acquisition (i.e., 20 ms or 50 Hz for current state-of-the-art sCMOS cameras in one-direction rolling shutter mode) and the use of long travel-range motorized sample stages for step-wise *z*-plane acquisition (typically > 50 ms motion and settling time, in addition to the camera exposure) which is generally the rate-limiting step. This results in a typical acquisition rate of 10–15 *z*-planes per second as reported previously [5]. ETLs are shown to easily achieve [33] > 30 Hz for the full-range coverage without any distortions, and they can achieve even higher speeds if run continuously using sinusoidal waveforms (given that the light sheets in LSTM have a significant confocal parameter, the synchronization of sinusoidal ETL waveforms with linear row-by-row detection is feasible). The ETLs can also be easily replaced with faster acoustic tunable lenses (e.g., TAG lenses from TAG Optics) for applications requiring much higher volumetric imaging speed (e.g., for the calcium imaging of functioning nervous systems). Hence, the introduction of ETLs in the LSTM imaging procedure has no consequences for the overall imaging speed *vis-à-vis* LSM for high-resolution imaging of large samples. We also found that, unlike LSM, LSTM does not require a pre-imaging calibration step for the estimation of sample-position-dependent alignment parameters, resulting in overall faster imaging speeds.

We demonstrated the LSTM performance by rapid high-resolution imaging of large samples of various sizes and shapes, including the entire intact mouse central nervous system, thick coronal sections of mouse and rat brains, a large chunk of human brain, uniformly expanded brain tissue, and a highly motile *Hydra*. Also, due to geometric advantage, LSTM is expected to enable volumetric calcium imaging in live rodent brains, similar to SCAPE [22], especially by combining with rapid de-focusing/focusing (e.g., using ETLs in the detection arm). We also performed direct comparative imaging of the same thick rat brain coronal section using LSTM and LSM (Fig. 6) to demonstrate that LSTM indeed eliminates the limit on lateral dimensions of imaging volumes while providing high imaging speed and uniform imaging resolution. Therefore, LSTM provides complementary advantages over LSM for rapid high-resolution imaging of very large samples. These capabilities of LSTM are expected to significantly accelerate our understanding of healthy and diseased tissue architectures. Future work will include integration of super-resolution approaches (such as structured illumination) and simultaneous multi-view imaging.

## Conclusions

We report the development of a distinct light sheet microscopy (LSM) approach, termed light sheet theta microscopy (LSTM), which addresses the fundamental limitation of LSM on the lateral dimensions of imaging volumes due to the orthogonal illumination-detection geometry. We have presented extensive characterization of the LSTM system properties and performed several real-world high-resolution imaging experiments of very large samples, including mouse and rat brains, a large section of human brain, a highly expanded expansion microscopy sample, and rapid volumetric calcium imaging of a highly motile *Hydra*. These new imaging capabilities will enable numerous novel applications, including imaging of an entire post-mortem human brain (thick slab-by-slab) in a practical time-frame and direct in situ transcriptomics of whole rodent brains.

## Methods

### LSTM implementation

The optical layout and physical implementation details are presented in Fig. 2, Additional file 1: Figure S1, and Additional file 2: Figure S2. A complete parts list is provided in Table 1. The illumination arms of the LSTM instrument consist of a laser source (Omicron-Laserage, Rodgau, Germany; SOLE-6 engine containing four wavelengths: 405, 488, 561, and 647 nm), a cylindrical lens (Thorlabs, Newton, NJ, USA; LJ1695RM-A, *f* = 50 mm), a vertical slit (Thorlabs, VA100C), an iris (Thorlabs, CP20S), an electrically tunable lens (Optotune, Dietikon, Switzerland; EL-16-40-TC), a galvo scanner (Thorlabs, GVS001), a scan lens (Thorlabs, CLS-SL), a tube lens (Thorlabs, ITL200), and the illumination objective (Olympus Macro 4×/0.28NA/28.5 mmWD air). Since the illumination objectives were air objectives, we used a 3D-printed cap (using Ultimaker 2 + extended, in polylactic acid (PLA)) containing a 1-in. diameter quartz coverslip, to allow dipping in immersion oil, ensuring that the low-numerical aperture (NA) light rays enter the media near perpendicularly. The detection arm is composed of a detection objective (Olympus, XLPLN10XSVMP/10× or XLSLPLN25XGMP/25×), a tube lens (Thorlabs, TTL200), a multi-band emission filter (Semrock, FF01-432/515/595/730-50-D), and an sCMOS camera (Hamamatsu Orca Flash 4.0 V3). The illumination arms were vertically mounted at an approximately 60° angle relative to the detection axis. To facilitate the optical alignment adjustments, all three optical arms were mounted on manual translation stages (Thorlabs, TBB0606) attached to the breadboard. We used a 3D-printed (using Ultimaker 2 + extended in PLA) open-top sample chamber that was filled with an immersion oil of refractive index 1.454 (Cargille Laboratories, Cedar Grove, NJ, USA). The CLARITY-cleared (refractive index = ~ 1.454) sample was mounted in a quartz cuvette (refractive index ~ 1.45, FireflySci, Staten Island, NY, USA) which was then affixed to appropriate grooves at the base of the sample chamber (Additional file 1: Figure S1). The 3D model of the LSTM microscope was made with Autodesk Inventor 2017.

### LSM imaging

All LSM imaging experiments were performed using the COLM implementation as described previously [5]. Briefly, the optical components were the same as described for LSTM, i.e., detection objectives: Olympus 10×/0.6NA/8mmWD or Olympus 25/1.0NA/8mmWD with a correction collar for the refractive indices of water to oil; sCMOS camera: Hamamatsu Orca Flash 4.0 V3; low-NA illumination objective: Olympus Macro 4×/0.28 NA; tube lens: Thorlabs TTL200; and scan lens: Thorlabs, CLS-SL. The dynamic light sheets are generated by rapid scanning of Gaussian beams (Thorlabs, GVS102). Similar to LSTM, COLM uses synchronized illumination-detection to improve the imaging quality. Additionally, an automated-alignment parameter calibration (using linear adaptation) corrects for misalignment artifacts across the whole sample space.

### LSTM geometric constraint calculations

The physical geometric constraints of the oblique arrangement of illumination and detection objectives were analyzed by calculating the upper and lower bounds (Fig. 3a, Additional file 3: Figure S3) with the following criteria: the illumination objective should not touch the detection objective (Fig. 3a top) and it should not extend below the physical extent of the detection objective (Fig. 3a middle). The range of allowable angular positions was calculated by taking the effective working distances (WDs) and the objective diameters into account as shown in the schematics of Additional file 3: Figure S3, resulting in the following relationships:

$$ W2\ast \sin \left(\uptheta i\right)=\frac{D1}{2}+D2\ast \frac{\cos \left(\uptheta i\right)}{2} $$

$$ W2\ast \cos \left(\uptheta f\right)=W1+D2\ast \frac{\sin \left(\uptheta f\right)}{2} $$

where *W*_1_ and *W*_2_ are the WDs of the detection and illumination objectives respectively, *D*_1_ and *D*_2_ are the diameters of the detection and illumination objectives respectively, and θ_i_ and θ_f_ are the boundary angular positions. Since we used a Macro 4×/0.28NA/29.5WD (Olympus) air objective for illumination, the approximate effective WDs were calculated as shown in Additional file 3: Figure S3. For most of the experiments we used a 10×/0.6NA/8mmWD objective (Olympus) in the detection arm with values of *W*_1_ = 8, and *D*_1_ = 40. For this combination, we found the allowable angular range to be 43.3^°^ to 62.3^°^, which served as our initial guide for identifying the maximum possible angular positioning. We used ~ 60^°^ as the final angle separation. All calculations were performed in MATLAB.

### Illumination depth calculations

We used geometric calculations (Fig. 3b) to estimate the maximum illumination path lengths (MIPLs) of LSTM as *t*/ cos(θ), where *t* is the sample thickness to be imaged and θ is the angle between the illumination propagation direction and the detection axis. The MIPL in LSM would be the same as the sample width (*w*). We calculated the ratio of these illumination path lengths, which was then converted into a binary representation by thresholding at 1 and plotted as a heat map, shown in Fig. 3b. The edge effects were approximated.

### LSTM effective light sheet thickness calculations

Due to the non-orthogonal incidence of the light sheets on the detection plane, the effective light sheet thickness can be approximated as the projection of the original thickness onto the detection direction, resulting in *b*/ sin(θ), where *b* is the actual light sheet thickness at the most focused position, and θ is the angle of incidence relative to the detection axis. The relationship is plotted in Fig. 3c.

### Imaging experiments

All the imaging experiments are summarized in Table 2. We used the passive CLARITY method (as described previously [5]) for all the tissue clarification experiments. The hydrogel monomer (HM) solution recipe consisted of 1–4% (wt/vol) acrylamide, 0.05% (wt/vol) bisacrylamide, 4% paraformaldehyde (PFA), 1× phosphate-buffered saline (PBS), deionized water, and 0.25% thermal initiation VA-044 (Wako Chemicals, Boston, MA, USA; NC0632395). All animal procedures were followed according to Institutional Animal Care and Use Committee (IACUC) guidelines. For whole brain clearing, transcardiac perfusion was performed with 20 mL HM solution, followed by overnight incubation at 4 °C. The rat brain was perfused with 4% PFA, post-fixed for 16 h, and then frozen in isopentane for storage. The frozen brain was thawed at room temperature in PBS buffer, then sliced and incubated in HM solution overnight at 4 °C. The human brain tissue was incubated in 4% PFA for ~ 2 days, followed by incubation in HM solution overnight at 4 °C. All the perfused tissues were de-gassed and then stored at 37 °C for 3–4 h for hydrogel polymerization. The tissues were cleared by incubating (with shaking) in clearing buffer (4% (wt/vol) sodium dodecyl sulfate (SDS), 0.2 M boric acid, pH 8.5) at 37 °C until clear (2–3 weeks). Afterwards, the tissues were washed with 0.2 M boric acid buffer (pH 8.5) with 0.1% Triton X-100 for up to 24 h. The cleared tissue was labeled with DAPI (1 μg/mL final concentration) and/or the blood vessel marker tomato lectin (Vector Labs, Burlingame, CA, USA; FL-1171) by incubating in the labeling solution for 3–4 days. After washing with the buffered solution (0.2 M boric acid buffer, pH 7.5, 0.1% Triton X-100), the tissue was transferred into 85–87% glycerol solution in graded fashion (i.e. 25%, 50%, 65%, and finally 87%) for final clearing and imaging. For uniform tissue expansion (4–4.5× uniformly), a *Thy1-eYFP* mouse brain slice (250 μm, perfused and fixed with 4% PFA and sliced with vibratome) was gelled and digested following the protein retention expansion microscopy (proExM) protocol [34]. The sample was stored in 1× PBS before changing the buffer to 65% glycerol (with 2.5 mg/mL 1,4-diazabicyclo[2.2.2]octane (DABCO)) for the LSTM imaging. All imaging experiments were performed with an effective light sheet thickness of 2–5 μm.

**Table 2:** Summary of imaging experiments reported in this study

Samples	Fig. No.	Label	Det. objective	Illum. objective	Imaging volume dimensions	No. of images/raw data	Imaging time
Thick human brain tissue	4	DAPI	10×/0.6NA/8mmWD	4×/0.28NA/28.5WD	~ 10.5 mm × 14.1 mm × 3 mm	116,736/~ 0.97 TB	~ 2.7 h
Mouse brain with attached spinal cord	5a	*Thy1-eYFP*	10×/0.6NA/8mmWD	4×/0.28NA/28.5WD	11.8 mm × 27.6 mm × 5.2 mm	388,687/~ 3.3 TB	~ 9 h
Thick mouse brain slice	5b	*Thy1-eYFP*	10×/0.6NA/8mmWD	4×/0.28NA/28.5WD	~ 9.6 mm × 13.5 mm × 5.34 mm	256,560/ ~ 2.1 TB	~ 5.9 h
Thick mouse brain slice	5c	*Thy1-eYFP*	25×/1.0NA/8 mm/WD	4×/0.28NA/28.5WD	6 mm × 9.6 mm × 1.9 mm^a^	211,616/ ~ 1.8 TB	~ 4.9 h
Thick rat brain slice	6a, b	Tomato lectin	10×/0.6NA/8mmWD	4×/0.28NA/28.5WD	~ 20 mm × 16.5 mm × 3.6 mm deep^b^	285,821 slices/2.4 TB	~ 6.6 h
expanded (~ 4×) mouse brain slice	6c	*Thy1-eYFP*	10×/0.6NA/8mmWD	4×/0.28NA/28.5WD	33.2 mm × 19.3 mm × 2 mm^c^	723,200/ ~ 6 TB	~ 22 h
*Hydra* live imaging	7	GCaMP6s	10×/0.6NA/8mmWD	4×/0.28NA/28.5WD	1.2 mm × 1.2 mm × 0.136 mm	23,001/~ 193 GB	~ 1 h live imaging

Summary of the datasets reported in this report.

^a^The image volume acquired was ~ 6 mm × 9.6 mm × 1.9 mm; however, due to constraints of high-quality volume rendering, a smaller (0.5-mm-thick) subset was used for the rendering shown in Fig. 5c

^b^The approximate imaging volume was ~ 20 mm × 16.5 mm × 3.6 mm, and a few ~ 5-mm-deep image stacks were acquired to demonstrate the imaging depth in Fig. 6b

^c^The imaging volume acquired was ~ 33.2 mm × 19.3 mm × 2 mm to ensure complete coverage of ~ 1-mm-thick expanded non-rigid tissue

*TB* terabytes, *GB* gigabytes, *h* hours

### Image analyses

A TeraStitcher [35]-based pipeline [5] was used for the stitching of acquired image stack tiles of all the datasets. Maximum intensity projections and other linear image contrast adjustments were performed using Fiji [36, 37] and MATLAB. All volume renderings were performed using Amira (FEI, Lausanne, Switzerland). All the fluorescent bead image analyses were performed using Fiji. To calculate the axial full width at half maximum (FWHM), *x*-*z* projections of the bead image stacks were used. For individual beads a line intensity profile was calculated along the central axis, followed by manual calculations of FWHM intensity values.

### Illumination energy load calculations

The procedure is summarized in Additional file 7: Figure S5. To calculate the total illumination energy load in LSTM, we performed a simulation of the step-wise scanning of the sample through the illuminating light sheet. A horizontal plane across an entire sample can be imaged with approximately non-overlapping thin sheets of light; therefore, the total energy is calculated by step-wise scanning of the sample through the illumination volume. All voxels receiving illumination are incremented by 1. The final energy load is calculated as the total sum of accumulated illuminations in all voxels. The procedure was implemented for a range of parameters and two detection objectives (10×/0.6NA/8mmWD and 25/1.0NA/8mmWD). For LSM calculations, each voxel is illuminated (*w*/*f*) times, where *w* is the width of the sample, and *f* is the field-of-view size of the detection arm. Therefore, the total energy load is approximated as (*w*/*f*)*number of voxels. Note that the energy load in LSM as well as LSTM scales up by the same constant factor, which cancels out in the ratio.

## Additional files

Additional file 1: **Figure S1.** LSTM microscopy implementation. (a) Image of the physical LSTM setup. (b) 3D model of LSTM and the sample mounting system. The 3D-printed sample chamber is designed to accommodate large biological samples of virtually any dimensions, while still allowing the objectives to be immersed in the immersion oil. Two transparent glass windows, located on the lateral sides, provide visual view of the sample for ease of positioning. An additional window is realized at the bottom part of the chamber to allow the illumination light to pass through. An additional adapter was designed to allow mounting a prism mirror at about approximately 10° from the normal surface to facilitate the optical alignment of the system. (PDF 1623 kb)

Additional file 2: **Figure S2.** Detailed annotation of LSTM optical path shown in Fig. 2a. (PDF 571 kb)

Additional file 3:**Figure S3.** Physical constraints of positioning illumination and detection objectives. (a) Schematics showing calculations of the elongated working distance (*EWD*) of the air illumination objective (Olympus Macro 4×/0.28NA/29.5WD Air) when used in immersion liquid (refractive index 1.454). Original working distance (*OWD*) is the working distance in air according to the objective specifications. A thin quartz coverslip and a 3D-printed cap were used to seal the illumination objectives. EWD was estimated to be 43.84 mm. (b) Geometric constraints calculation for the co-arrangement of the illumination and detection objectives. The two boundary conditions are shown in *blue* and *green* shading of the illumination objective. For the upper bound limit (*blue*), the relationship among different parameters is defined by the equation $W2\ast \sin \left(\uptheta i\right)=\frac{D1}{2}+D2\ast \frac{\cos \left(\uptheta i\right)}{2}$. For the lower bound limit (*green*), it is defined by $W2\ast \cos \left(\uptheta f\right)=W1+D2\ast \frac{\sin \left(\uptheta f\right)}{2}$. *W*_1_ and *W*_2_ are the effective working distances of detection and illumination objectives respectively. *D*_1_ and *D*_2_ are the diameters of the detection and illumination objectives respectively. θ_i_ and θ_f_ are the angular positions of upper and lower bounds respectively. For the 4×/0.28NA/29.5 mmWD (as illumination objective) and 10×/0.6NA/8mmWD (as detection objective), the calculated θ_i_ and θ_f_ are 43.32° and 63.37° respectively. This range served as a starting point during the optical alignment of the system. (PDF 596 kb)

Additional file 4: **Video 1.** 3D model of LSTM implementation. The 3D modeling and rendering was performed using Autodesk Inventor 2017, and the animation was performed using Autodesk Fusion 360 2017 and MATLAB. The components labeled are *LS* (laser source), collimator, *ND* (neural density) filter mount, iris, *ETL* (electrically tunable lens), slit, *CL* (cylindrical lens), galvo scanner, *SL* (scan lens), iris and *TL* (tube lens). The high-resolution video is available in the figshare repository at https://doi.org/10.6084/m9.figshare.c.4072160. (MP4 82944 kb)

Additional file 5: **Video 2.** Comparison of image volumes acquired with LSTM in 1-axis scan (*1-AS*) and 2-axis scan (*2-AS*) modes. The 3D rendering visualizes the image stacks acquired from the same sample (human brain section stained with DAPI) with LSTM in 1-AS and simultaneous 2-AS modes. The high-resolution video is available in the figshare repository at https://doi.org/10.6084/m9.figshare.c.4072160. (MOV 104448 kb)

Additional file 6:**Figure S4.** Comparison of maximum illumination path lengths in LSTM and LSM. The graphs plot the binarized ratios ($w/\left(\frac{t}{\cos \left(\uptheta \right)}\right)$) of maximum illumination path lengths required for complete coverage of samples of various widths (*w*) and thicknesses (*t*) for different angular arrangements. *Magenta* and *cyan* regions mark the combinations of *w* and *t* for which the illumination path lengths were smaller in LSTM and LSM, respectively. (PDF 262 kb)

Additional file 7: **Figure S5.** Total illumination energy load in LSTM vs. LSM. The schematic summarizes the calculations of total energy loads imparted in LSTM and LSM for imaging of a sample of specific dimensions, imaged with a specific detection objective. (a) In LSTM, a horizontal plane across the entire sample is imaged with approximately non-overlapping thin sheets of light. Therefore, total energy load can be calculated by step-wise scanning of the sample (for each plane) through the illuminating light. For each of the steps, all voxels that receive light are incremented by 1. The procedure was implemented for a range of parameters and two detection objectives (10×/0.6NA/8mmWD and 25/1.0NA/8mmWD). (b) In LSM a stack (or tile) is acquired by approximately non-overlapping thin sheets of light. The total energy load is calculated by summing up the illumination for all tiles in a row along the width. Note that the dwell time of illumination line profile is same for both LSTM and LSM (scanned light sheet implementation, e.g., COLM). The energy load for tiles along the sample length scales up by the same constant factor in LSTM and LSM; therefore, we only simulated one row of tiles along the sample width. (PDF 556 kb)

Additional file 8: **Video 3**. High-resolution LSTM imaging of intact *Thy1-eYFP* mouse central nervous system. The bounding box for the entire sample is 11.8 mm × 27.6 mm × 5.2 mm, and for the subvolume shown is 5.1 mm × 3.1 mm × 3.5 mm. The raw data was down-sampled 2 × 2 fold (to make the volume rendering feasible) for the subvolume rendering. The high-resolution video is available in the figshare repository at https://doi.org/10.6084/m9.figshare.c.4072160. (MOV 143360 kb)

Additional file 9: **Video 4.** High-resolution LSTM imaging of a large tissue of *Thy1-eYFP* mouse brain. The bounding box is 9.6 mm × 13.5 mm × 5.34 mm. The raw data was down-sampled 4 × 4 fold to make the volume rendering feasible. The high-resolution video is available in the figshare repository at https://doi.org/10.6084/m9.figshare.c.4072160. (MOV 167936 kb)

Additional file 10: **Video 5.** Visualization of an image stack of vasculature-stained rat brain tissue. This video visualizes an image stack acquired from a large rat brain slice (stained for vasculature with tomato lectin) using LSTM in 2-AS mode. The bounding box is 1 mm × 1 mm × 5 mm. The high-resolution video is available in the figshare repository at https://doi.org/10.6084/m9.figshare.c.4072160. (MOV 143360 kb)

Additional file 11: **Video 6.** High-resolution LSTM imaging of a large expanded section of *Thy1-eYFP* mouse brain. A thin (250 μm) coronal section was expanded ~ 4-fold using proExM procedure and imaged using LSTM with 10×/0.6NA detection objective. The resulting dataset (~ 6 TB) consisted of 723,300 full frame images (2048 × 2048). The data was down-sampled 8 × 8 fold to allow high-quality volumetric rendering. The high-resolution video is available in the figshare repository at https://doi.org/10.6084/m9.figshare.c.4072160. (MOV 439296 kb)

Additional file 12: **Video 7.** Rapid volumetric calcium imaging of highly motile *Hydra*. GCaMP6s-expressing *Hydra* was imaged using LSTM with 10×/0.6NA objective. Maximum intensity projections are shown for the two halves of the volume. First occurrences of longitudinal and radial contractions are annotated. The scale bar is 100 μm. The high-resolution video is available in the figshare repository at https://doi.org/10.6084/m9.figshare.c.4072160. (MOV 66662 kb)

Additional file 13: **Video 8.** Neuronal activity traces of representative neurons. A visualization of the neuronal traces shown in Fig. 7b. The high-resolution video is available in the figshare repository at https://doi.org/10.6084/m9.figshare.c.4072160. (MOV 11161 kb)
