# Supplementary figures and images for: Light sheet theta microscopy for rapid high-resolution imaging of large biological samples
Source: BMC Biol. 2018 May 29;16:57. doi: 10.1186/s12915-018-0521-8 (PMC5975440; doi:10.1186/s12915-018-0521-8)

a

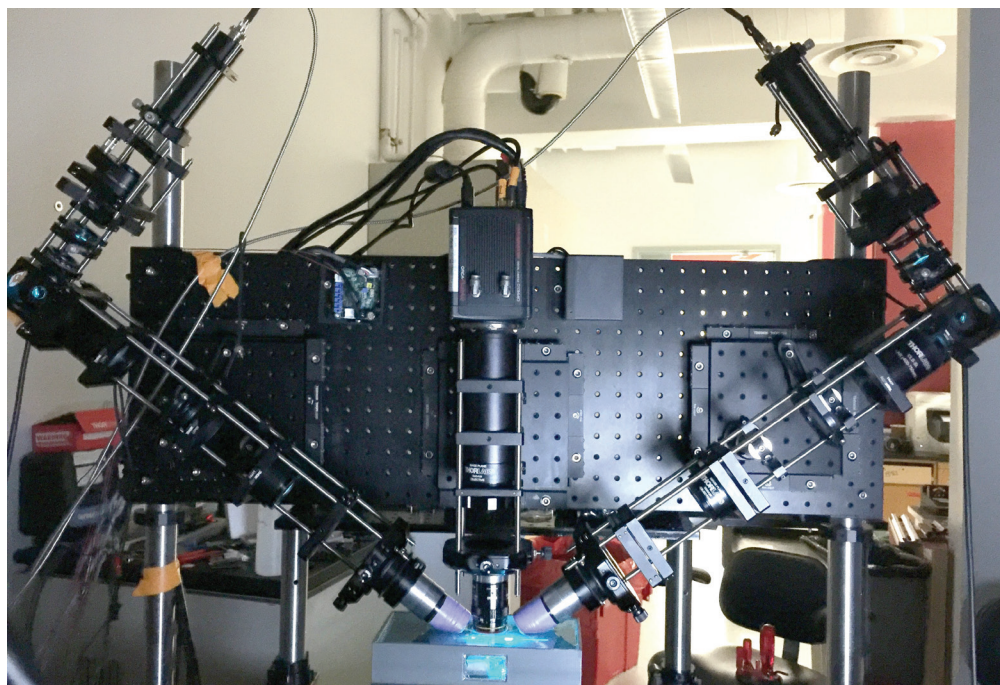

b

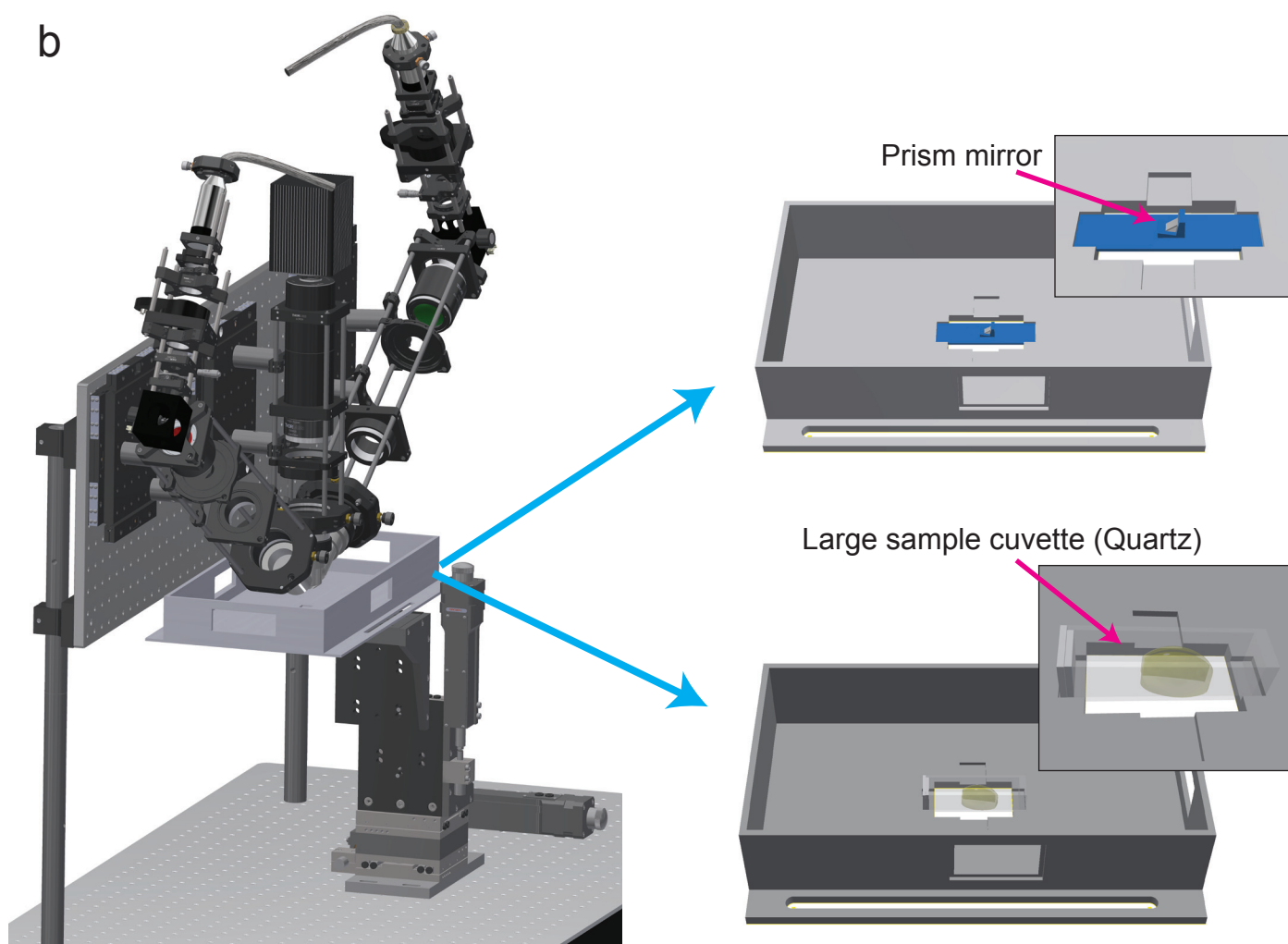

Supplement: Supplementary file 1 — Figure S1. LSTM microscopy implementation. (a) Image of the physical LSTM setup. (b) 3D model of LSTM and the sample mounting system. The 3D-printed sample chamber is designed to accommodate large biological samples of virtually any dimensions, while still allowing the objectives to be immersed in the immersion oil. Two transparent glass windows, located on the lateral sides, provide visual view of the sample for ease of positioning. An additional window is realized at the bottom part of the chamber to allow the illumination light to pass through. An additional adapter was designed to allow mounting a prism mirror at about approximately 10° from the normal surface to facilitate the optical alignment of the system. (PDF 1623 kb) [file 12915_2018_521_MOESM1_ESM.pdf]

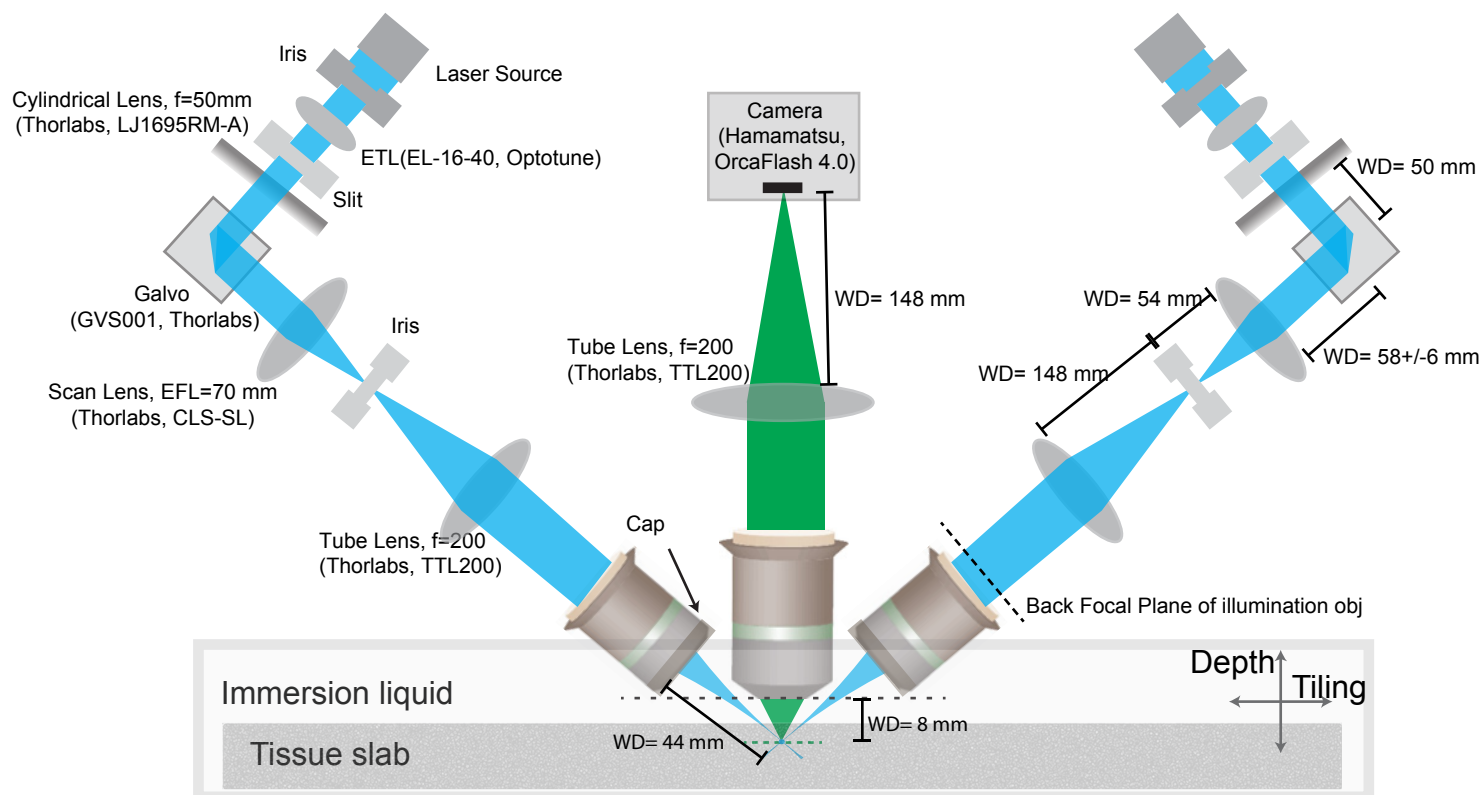

Supplement: Supplementary file 2 — Figure S2. Detailed annotation of LSTM optical path shown in Fig. 2a. (PDF 571 kb) [file 12915_2018_521_MOESM2_ESM.pdf]

a

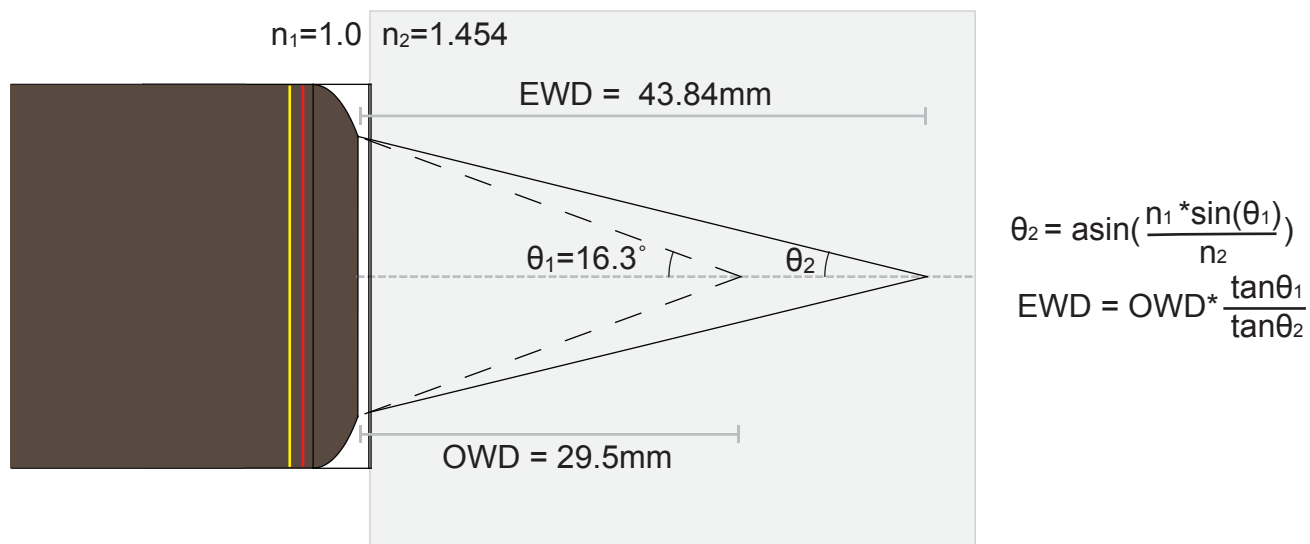

b

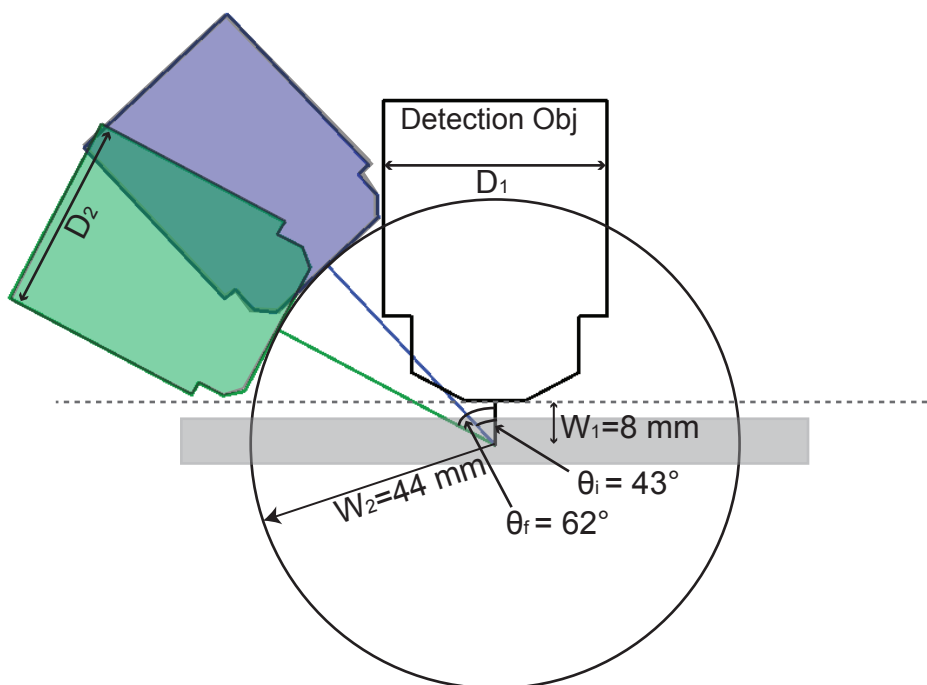

Supplement: Supplementary file 3 — Figure S3. Physical constraints of positioning illumination and detection objectives. (a) Schematics showing calculations of the elongated working distance (EWD) of the air illumination objective (Olympus Macro 4×/0.28NA/29.5WD Air) when used in immersion liquid (refractive index 1.454). Original working distance (OWD) is the working distance in air according to the objective specifications. A thin quartz coverslip and a 3D-printed cap were used to seal the illumination objectives. EWD was estimated to be 43.84 mm. (b) Geometric constraints calculation for the co-arrangement of the illumination and detection objectives. The two boundary conditions are shown in blue and green shading of the illumination objective. For the upper bound limit (blue), the relationship among different parameters is defined by the equation $W2\ast \sin \left(\uptheta i\right)=\frac{D1}{2}+D2\ast \frac{\cos \left(\uptheta i\right)}{2}$W2∗sinθi=D12+D2∗cosθi2. For the lower bound limit (green), it is defined by $W2\ast \cos \left(\uptheta f\right)=W1+D2\ast \frac{\sin \left(\uptheta f\right)}{2}$W2∗cosθf=W1+D2∗sinθf2. W1 and W2 are the effective working distances of detection and illumination objectives respectively. D1 and D2 are the diameters of the detection and illumination objectives respectively. θi and θf are the angular positions of upper and lower bounds respectively. For the 4×/0.28NA/29.5 mmWD (as illumination objective) and 10×/0.6NA/8mmWD (as detection objective), the calculated θi and θf are 43.3 [file 12915_2018_521_MOESM3_ESM.pdf]

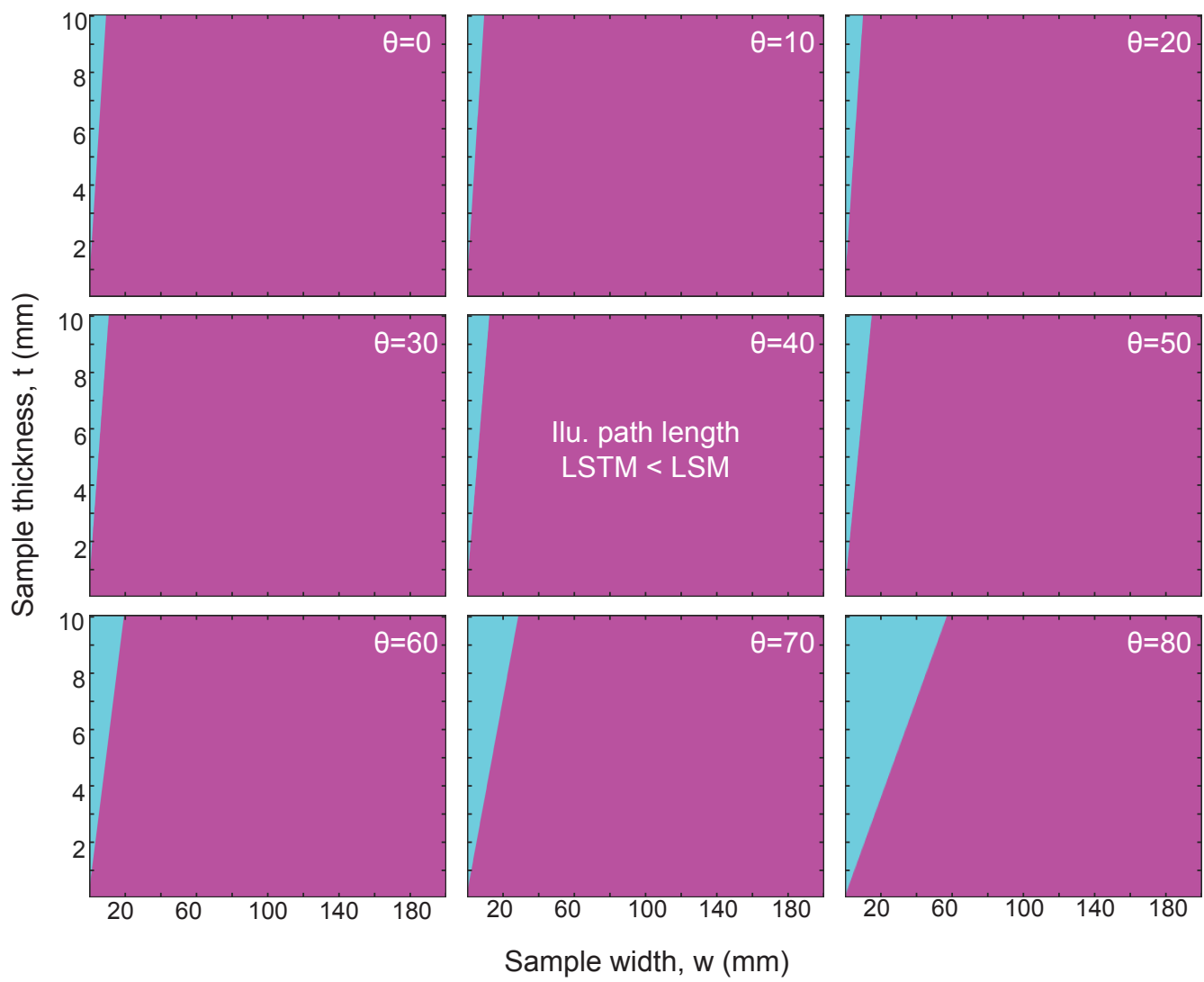

Supplement: Supplementary file 6 — Figure S4. Comparison of maximum illumination path lengths in LSTM and LSM. The graphs plot the binarized ratios ($w/\left(\frac{t}{\cos \left(\uptheta \right)}\right)$w/tcosθ) of maximum illumination path lengths required for complete coverage of samples of various widths (w) and thicknesses (t) for different angular arrangements. Magenta and cyan regions mark the combinations of w and t for which the illumination path lengths were smaller in LSTM and LSM, respectively. (PDF 262 kb) [file 12915_2018_521_MOESM6_ESM.pdf]

## a Total energy load in LSTM imaging

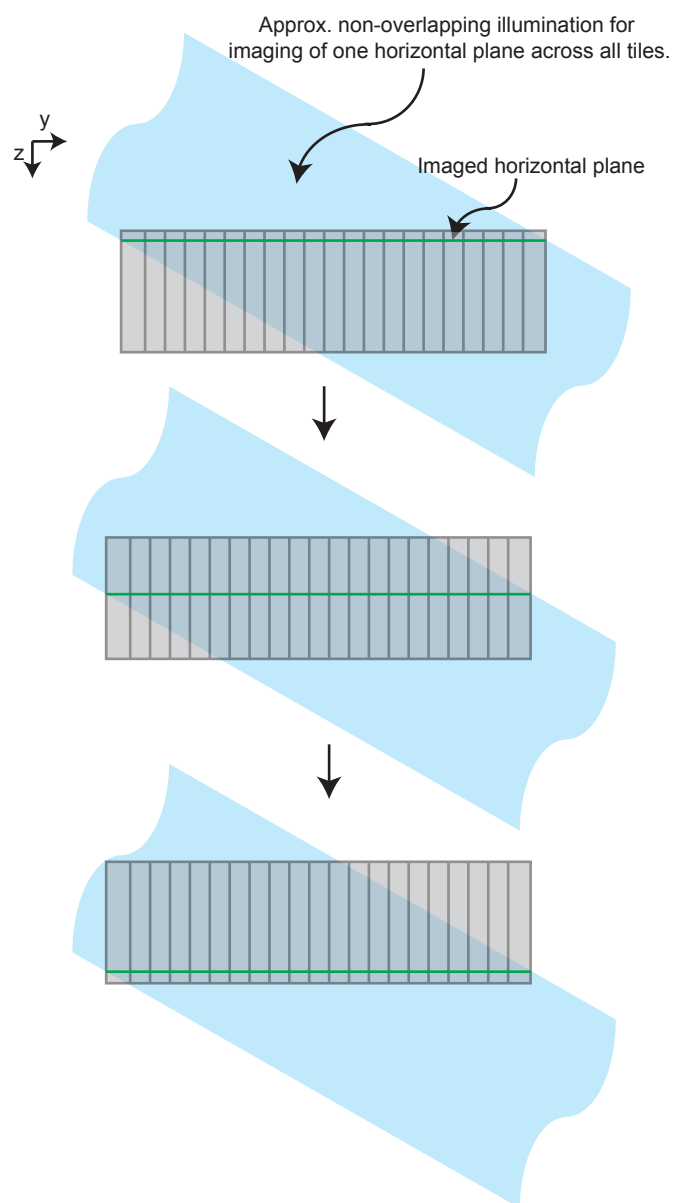

## b Total energy load in LSM imaging

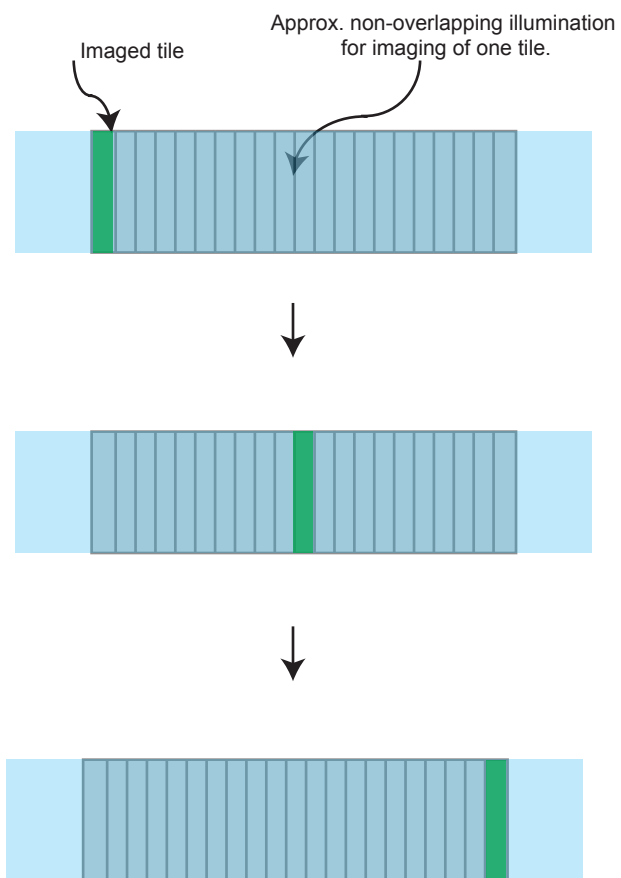

Supplement: Supplementary file 7 — Figure S5. Total illumination energy load in LSTM vs. LSM. The schematic summarizes the calculations of total energy loads imparted in LSTM and LSM for imaging of a sample of specific dimensions, imaged with a specific detection objective. (a) In LSTM, a horizontal plane across the entire sample is imaged with approximately non-overlapping thin sheets of light. Therefore, total energy load can be calculated by step-wise scanning of the sample (for each plane) through the illuminating light. For each of the steps, all voxels that receive light are incremented by 1. The procedure was implemented for a range of parameters and two detection objectives (10×/0.6NA/8mmWD and 25/1.0NA/8mmWD). (b) In LSM a stack (or tile) is acquired by approximately non-overlapping thin sheets of light. The total energy load is calculated by summing up the illumination for all tiles in a row along the width. Note that the dwell time of illumination line profile is same for both LSTM and LSM (scanned light sheet implementation, e.g., COLM). The energy load for tiles along the sample length scales up by the same constant factor in LSTM and LSM; therefore, we only simulated one row of tiles along the sample width. (PDF 556 kb) [file 12915_2018_521_MOESM7_ESM.pdf]
